# Critically engaging: integrating the social and the biomedical in international microbicides research

**DOI:** 10.1186/1758-2652-14-S2-S4

**Published:** 2011-09-27

**Authors:** Catherine M Montgomery, Robert Pool

**Affiliations:** 1 Department of Sociology, University of York, UK; 2 Barcelona Centre for International Health Research (CRESIB), University of Barcelona, Spain; 3 Centre for Social Science and Global Health, University of Amsterdam, The Netherlands

## Abstract

Randomized controlled trials and critical social theory are known not to be happy bedfellows. Such trials are embedded in a positivist view of the world, seeking definitive answers to testable questions; critical social theory questions the methods by which we deem the world knowable and may consider experiments in the biomedical sciences as social artifacts. Yet both of these epistemologically and methodologically divergent fields offer potentially important advances in HIV research. In this paper, we describe collaboration between social and biomedical researchers on a large, publicly funded programme to develop vaginal microbicides for HIV prevention. In terms of critical engagement, having integrated and qualitative social science components in the protocol meant potentially nesting alternative epistemologies at the heart of the randomized controlled trial. The social science research highlighted the fallibility and fragility of trial data by demonstrating inconsistencies in key behavioural measurements. It also foregrounded the disjuncture between biomedical conceptions of microbicides and the meanings and uses of the study gel in the context of users’ everyday lives. These findings were communicated to the clinical and epidemiological members of the team on an ongoing basis via a feedback loop, through which new issues of concern could also be debated and, in theory, data collection adjusted to the changing needs of the programme. Although critical findings were taken on board by the trialists, a hierarchy of evidence nonetheless remained that limited the utility of some social science findings. This was in spite of mutual respect between clinical epidemiologists and social scientists, equal representation in management and coordination bodies, and equity in funding for the different disciplines. We discuss the positive role that social science integrated into an HIV prevention trial can play, but nonetheless highlight tensions that remain where a hierarchy of epistemologies exists alongside competing paradigms and priorities.

## Background

Hailed as the gold standard in clinical evaluation, the randomized controlled trial (RCT) has become a central feature of the development of new drugs and medical devices. As May has noted, the symbolic capital of the clinical trial arises in part from the purity of the design and associated scientific rigour; the RCT is the touchstone of clinical epidemiology, and the standard against which other research and reputations are measured [1]. Indeed, underscoring its privileged place within the medical sciences, it has been said in relation to the RCT that “our ability to evaluate rigorously what we do clinically remains the essence of modern biomedicine” [2]. This is nowhere more true than in the field of HIV treatment and prevention [3].

The RCT is posited as the ideal experiment. Placebo control, blinding and randomization - enshrined in rigorous trial protocols that get imposed on everyday life and clinical practice - are designed to minimize social contingencies and allow the objective, a-contextual measurement of reality. Methods are based on a positivist paradigm that assumes the existence of a single, fixed reality and the possibility of neutral, objective and value-free knowledge of that reality. It is these measurement ideals, embodied in RCT methodology, that assure the RCT its hallowed place in medical research, at the very heart of evidence-based medicine (EBM).

However, it is these very same ideals and methodological assumptions that lead some social scientists to critique the RCT (and EBM more broadly), arguing that it relies on human decisions about classification that are deeply embedded in cultural conventions. Thus it has been argued that the RCT does not simply describe external reality, but actively helps to create it [4,5]. This view is based on constructivist and interpretivist paradigms that assume multiple, context-dependent and historically contingent realities, influenced or produced by the values of the researcher and by the research process itself (for an overview of EBM in relation to social science critiques, see [6]).

Such fundamental differences in ontology and epistemology have been seen to map unproblematically onto the medical and the social sciences. The presumed antipathy between the two has spawned numerous critical assessments of collaboration in both directions (epitomized by the so-called “paradigm wars” [7]), with the social sciences calling into question the credibility and objectivity of RCT evidence, and medical researchers deeply suspicious of what counts as evidence in the social sciences. For example, Pareja Béhague *et al* report that anthropological work is often regarded as soft, anecdotal and biased by virtue of the fact that it is based on small numbers of purposively selected informants. It is therefore deemed subjective rather than scientific [8]. Furthermore, while social scientists working with medical researchers may be expected to be conversant with epidemiological concepts and terminology, reference to social theorists among clinical epidemiologists is likely to be met with blank faces. Against this backdrop, Napolitano and Jones propose that “those belonging to the culture of social science and those belonging to the culture of medicine still appear to exist in almost totally separate conceptual universes” [9].

But how monolithic is the distinction between social and biomedical researchers? While the RCT itself embodies a regulated and standardized structure of evidence making, collaborators on a trial may espouse a variety of philosophical and methodological positions. Diversity may exist not only between biomedical and social researchers, but also between social scientists and among qualitative researchers [10,11]. For example, a wide range of theoretical and methodological views are represented by people defining themselves as social scientists. Many social scientists, including some ethnographers, use positivist methods and approaches. Some social scientists using quantitative methods adopt a critical, non-positivist approach to their methods and data; see for example [12]. This suggests the potential for successful collaboration between disciplinary teams working on HIV trials. It also suggests, however, that bridging the social and the biomedical does not concern a simple dichotomy but a more nuanced set of tensions that traverse epistemological, methodological and disciplinary dispositions.

The negotiation of these tensions grows ever more pertinent; in recent years, it has not only become increasingly acceptable to include social science in HIV trials, but there is great demand for mixed-method research from biomedical researchers and funders alike. There are various reasons for this. HIV interventionists want to be able to change beliefs, attitudes and behaviours contributing to new infections, and medical researchers need to ensure that people are motivated to enrol in trials, adhere to regimens and procedures, and are retained. Data on adherence and acceptability are essential: it is pointless developing and testing an intervention or new drug if people end up not wanting to use it. These are all inherently sociological issues that require a more qualitative approach, and an awareness of this has developed in medical research settings over the past 20 years.

In this paper, we describe collaboration between social and biomedical researchers on a large, publicly funded programme to develop vaginal microbicides for HIV prevention. Although the term “social science” can encompass many different disciplines, from economics to psychology, we use it here in a more limited sense to refer to the study of the social world using anthropological and sociological methods and perspectives. Our own intellectual position is located broadly within social constructivism and critical anthropology, and it is these traditions that inform the discussion that follows. Constructivism refers to the idea that reality is not “out there” waiting to be discovered, but is actively produced - “constructed” - by those participating in it. For a full account of the varieties of constructivism, see Holstein and Gubrium [13].

### Integration: an empirical example

Our case study is the Microbicides Development Programme’s MDP 301 phase III trial of the candidate microbicide PRO 2000. MDP 301 was a randomized, double-blind, placebo-controlled trial to test the safety and efficacy of PRO 2000 for the prevention of vaginally acquired HIV infection. The trial recruited 9385 women at six sites in Uganda, Tanzania, Zambia and South Africa and randomized them to receive either placebo or PRO 2000 gel. Women were asked to insert an applicator of gel within one hour before every act of sexual intercourse. They received regular HIV testing and counselling, promotion of safer sex practices, free condoms and diagnosis and treatment of sexually transmitted infections. Details of the trial’s methodology and results have been published elsewhere [14,15].

Early in the trial design phase, a decision was made to include a substantial social science component that would assess and contribute to the accuracy of the clinical data on sexual behaviour, gel adherence and condom use [16,17]. The social science component also involved assessing product acceptability and participants’ comprehension of the study, including informed consent procedures.

The main source of data for the trial outcome was the case record form (CRF), a closed-response questionnaire administered in the clinic. On this, women were asked to report their sexual behaviour, such as the number of times they had sex in the previous week and the number of times they used the gel or a condom. At each site, approximately 100 women were additionally randomized to take part in social science procedures (7.7% of trial participants); this included completing pictorial coital diaries at home and taking part in in-depth interviews, which elicited reports of the same behaviours over the same time period as the CRF and coital diaries. During these interviews, women were also asked about any discrepancies in their reports of their sexual behaviour on the different instruments [16,17].

### Measuring behavioural outcomes: shifting meaning of key terms and behaviours

The mixed-method approach was based on the assumption that it would be possible to match data from the same women for the same time period collected using different research tools. Any discrepancies would be discussed with participants and, hopefully, resolved. Early on, it became clear that there were substantial discrepancies between data from the different sources, mainly relating to the reporting of numbers of sex acts. At the end of the trial, there were inconsistencies in 60% of the data (predominantly CRF data), though most of these (80%) were resolved during discussion with participants [16]. This process highlighted the fallibility and fragility of the CRF data, the main data for the trial.

Analysis of the in-depth interviews revealed reasons for much of the inaccuracy: misunderstandings, recording errors and forgetfulness. However, other factors emerged. Various categories that were important to the trial in terms of measuring adherence to study product and the proportion of exposures to HIV covered by the gel or a condom, and which were assumed to be constant and universal, turned out to vary between study locations and individuals or to be ambiguous and to change for participants as they were questioned about them. For example, structured questions about time periods focused on “days”, “weeks” and “months” on the assumption that these terms meant the same thing to researchers and participants across the different locations and through time. However, in practice, “the last month” could be interpreted as the last 30 days, or what remains of this calendar month, or the time since the last clinic visit, and “the last week” could mean the last seven days, or the period since last Monday. The lived reality of “day” always exceeded attempts to define it; units of time in the context of sexual practice cannot easily be measured by the clock, but are embedded in social interactions, in artefacts, in the body, and in the environment [18]. This is starkly illustrated by the Swahili day, which starts at 6am and not when the clock turns midnight.

Perhaps even more central to the outcome of the trial was the category of “sex act”. Whereas we all know what we mean when we say we have “had sex”, this becomes complicated when we attempt to clearly define what an “act of sex” is and what separates one sex act from another. The complexity increases when working across multiple socio-cultural and linguistic settings, where, for example, sex may be referred to as “meeting” your partner and people distinguish between “sex acts” and “rounds”. The trial issued the following definition of a “sex act”: “penetrative vaginal sex that may or may not end with ejaculation”. In order to collect standardized and comparable data, interviewers across all settings were required to provide the same definition and ask the question in the same way throughout the trial. The assumption was that there is a single, fixed reality that could be measured in a universal and standardized way.

However, as the trial progressed and participants became familiar with this definition, the meaning of the questions about sex acts (and days and weeks) changed for them. What they reported as “one sex act” at the start of their participation was not necessarily the same as what they reported after months of iteration about the trial’s definition of sex. So divergent behavioural realities and temporal framing were brought within the logic of the structured trial CRF and its categories. Clearly, from a trial perspective, this had implications for the reliability and validity of data on the numbers of sex acts, but the RCT (and quantitative research more generally) does not allow for flexibility in the wording of questions to record these different realities and take into account their shifting meanings.

Because of the rigidity of RCT design, the “messy” data on inconsistencies emerging from the qualitative methods could not be used to adjust the main dataset used for analysis of the trial result. In other words, knowledge of the social circumstances of trial data production were excluded, even where the cost was a less “accurate” version of trial participants’ reported experiences. Collaboration between social and biomedical scientists over the measurement of sexual behaviour was therefore productive but limited; at the outset, it improved the validity of trial instruments, but it did not have any significant impact on the main trial data or its interpretation.

At the same time that divergent behavioural realities were being reclassified by participants and standardized by trial instruments, the behaviours themselves were also changing and adapting to the trial. Quite apart from changes requested by the trial as a condition for participation, there is evidence that, for many participants, the frequency of sex increased due to the increased sexual enjoyment and novelty resulting from gel, and there was experimentation, with some participants talking of experiencing a second youth. It is also likely that the repeated emphasis on the trial definition of sex will have had an impact on the reality of sex.

Sexual cultures are dynamic, they change naturally, and they change in response to research. Clinical trial research presumes a static field, dimensions of which can be measured but do not change, whereas social theory suggests that the mess and fluidity of the world can never be wholly captured by our attempts to pin it down, as well as the productive role of method itself in creating alterity:

“Practices generate that which they generate, but they also generate Otherness: homogeneity and heterogeneity; consistency and inconsistency; mensurability and uncountability; that which can be pinned down and delineated and that which cannot; grids and fluids. Residues and resistances are not technical failures - though it is possible to imagine them in this way. Rather they are intrinsic to practice, to being, which is, as it were, always more complex than it says or it can know” [19].

Critical, qualitative social science integrated into a clinical trial can both minimize the mess and draw attention to it. By revealing the large numbers of inconsistencies in the dataset upon which the trial result was based, social science drew attention to the “hidden work” of accomplishing an RCT that usually goes unremarked [20,21]. Such insights can be productive, but also pose a challenge, since underscoring the social contingencies of the RCT potentially threatens its very existence.

### Renewal and production: adjusting the lens

So far, we have discussed how issues of measurement were a central tension and site of production at the intersection of the social and the biomedical. Qualitative social science and epidemiology both offer approaches and styles of thought that direct one to particular questions and analytical foci. This cross-fertilization led to interesting and unanticipated research findings that enriched the trial and our knowledge of HIV dynamics. As Bryman observes, “Research of all kinds has the capacity to offer surprising or unexpected findings, but when quantitative and qualitative research are combined the possibilities of unplanned or unanticipated outcomes are magnified considerably” [7].

Under influence of the trialists’ need for representative and quantifiable data, the social science team increased sample sizes well beyond those normal for qualitative studies and used random selection, thus making the qualitative findings generalizable to the whole trial population. Also, the use of comparison groups made it possible to notice, and encouraged the explanation of, marginalized or stigmatized behaviours that were relevant for the trial but might not have arisen in a small convenience sample. For example, anal sex and multiple concurrent partners for women arose as new analytical domains for the social scientists, based on observations from the trial’s large main dataset.

Whereas the trial, by design, took an interest in the aggregate, integrated qualitative research could simultaneously explore the personal, delving into the nuances and complexity of lived social reality. Similarly, the tendency to conduct sub-group analyses in large quantitative data sets focuses analytical attention on questions that might not otherwise arise. For example, database reports showed that at one site, trial participants were reporting many more adverse events than participants at the other sites. Given that there was no biomedical rationale for this, it was assumed to be an artefact of the research process, but the question remained as to why it existed at this site. The social science team therefore decided to look at patterns of symptom reporting and the culture of medical research in different sites, topics that may not have arisen without the epidemiological rationale of doing so in this context.

If the epidemiology could be said to direct the line of sight, the social science suggested the lens through which data was collected. As far as possible, this lens was that of the people participating in the research rather than that of the people who had designed it. Typically in biomedical HIV research, questions are posed using predetermined categories based on the views of researchers, and uniform concepts transposed to a variety of geographical and cultural settings. Whereas this framing is presumed by biomedical researchers to be objective, or aperspectival (“a view from nowhere” as Goldenberg [4] puts it), anthropology refers to this view as the “etic” perspective. An “emic” approach, by contrast, enables us to take account of local meanings in a given cultural context by incorporating the conceptual schemes and categories that are meaningful to the study participants [22]. This approach proved particularly valuable in terms of understanding acceptability, both of trial procedures and study product.

For example, whereas microbicide acceptability has traditionally been framed in terms of HIV risk and individual behaviour change [23], we found that discourses of sexual pleasure and sexual health more broadly were at the fore of women and men’s accounts of use [24]. The social science foregrounded the disjuncture between biomedical conceptions of microbicides and the meanings and uses of gel in the context of users’ everyday lives. Although trial participants did not reject biomedical knowledge outright, they situated this in terms of their own experiences, cultural understandings and norms, such that the microbicide was conceptualized and employed in ways unimagined by drug developers or the medical researchers [25].

Similarly, in terms of the acceptability of the trial and its procedures, community response was couched in terms unlikely to have been captured by closed-response questionnaires, involving, at some sites, rumours of blood stealing and Satanism [26]. Social science research allowed these rumours to be interpreted and addressed so that threats to recruitment and retention could be minimized. Initial feedback from the social science data resulted in tailored community outreach, including, for example, invitations to observe clinical waste (including blood product) incineration; post-factum interpretations can inform future dialogue with communities whose members take part in clinical trials.

### Communication and compromise across epistemic and methodological divides

In MDP, the relationship between social scientists and clinical trialists was complex and ambivalent. On the one hand, there was a relative degree of equality and mutual respect between clinical epidemiologists and social scientists, equal representation in trial management and coordination bodies, and equity in funding. The very fact that a group of clinical trialists agreed to include a large qualitative, and potentially critical, social science component to such a degree in an important trial was without precedent. Trialists contributed substantially to the mixed-method design of the social science component, engaged in cross-disciplinary debate, and showed a striking and unusual degree of reflexivity and critique toward the assumptions underlying the trial methodology. Interdisciplinary communication was key to sustaining meaningful and productive collaboration, and occurred at all levels: within individual clinical sites, between clinical and coordinating sites, and among social science teams from different clinical sites. Regular interdisciplinary meetings, both face to face and via teleconference, brought social and biomedical concerns to the table simultaneously.

On the other hand, this collaboration involved compromises and adjustments that were largely on the side of the social scientists. We pursued an agenda of improving the trial, which entailed submitting to the cultural logic of the RCT and establishing a multi-level feedback loop to communicate with the different disciplinary teams. By “cultural logic” we refer to the requirements of the protocol, Good Clinical Practice and Institutional Review Boards (IRBs), which sometimes conflicted with the assumptions of social science and qualitative research. So, for example, while discussing the fluidity of the social world and the need for flexibility in its measurement, in practice we had to agree to limit adjustments to the in-depth interview guide because this would require new IRB approval. In another example, we felt that recording a sample of CRF interviews could help improve the data collection process, but getting permission from the trial to do this was fraught with lengthy debate about protocol amendments and new forms of consent for research staff conducting the interviews.

Priorities diverged not only at the point of data collection but also in expectations about analysis. Whereas we saw data collection and interpretation as coextensive, and presumed the need for flexibility to adjust the analytical lens, the trial required a fixed analysis plan upfront. This was driven by the desire to reduce bias and weed out the researcher’s subjective response or individual discretion in relation to the data. Whereas we acknowledged, and indeed explicitly incorporated, the researcher, this was anathema to clinical trialists, who aimed, and subsequently presumed themselves, to be absent from the data. Collaboration involved constant attempts by each party to assert these principles; social science analysis plans were usually only presented in “draft” form.

Finally, the two sides were not monolithic either, or rather, there were not so much two sides - clinical trial and social science - but a range of views that largely, but not exclusively, coincided with the disciplinary boundaries. Although most trialists were positivistically oriented, some had, or developed, relatively critical and reflexive perspectives, and the views among the social scientists ranged from critical anthropological to positivistic and applied. In this range of views, the centre of gravity was towards the positivistic rather than the critical-reflexive end. As a result, the integration of social science in the trial, although productive, could be seen, from a critical perspective, to have been more administrative than substantial: social science findings were not taken into account in the main trial results and the social science had no influence in the standard way in which the RCT was conducted.

On the other hand, many of the insights from the social science only emerged during the trial, and were accepted partly as a result of persistent lobbying during internal meetings, and so could not have been taken into account in the design of the trial. It is worth noting that the proposal for a second MDP trial has replaced the standard CRF for behavioural and adherence data with a revised version of the main qualitative in-depth interview tool from this trial.

## Discussion

In this paper, we have described the pragmatic approach to combining the social and the biomedical in MDP microbicides research. In summary, social science was used in four key ways:

1 Confirmation: we used triangulation of results from different methods to validate key behavioural measurements.

2 Complementarity: we used qualitative methods to elaborate, illustrate and clarify results from the quantitative methods, and quantitative data to critically reevaluate the qualitative data, thus aiding interpretation of the trial.

3 Development: we used results from the quantitative methods to inform qualitative enquiry and vice versa, including the design of tools, sampling of research subjects and substantive areas of enquiry.

4 Critique: we used social science data and extended observation of the functioning of the trial to constructively critique the standard RCT approach.

Clinical trials embody a search for coherence based upon a notion of stable human subjects, stable biotechnologies and predictable clinical outcomes. The reality is less clean cut: human subjects are not static in their behaviours, biotechnologies are not fully predetermined, and clinical outcomes may be predictable but are also the product of multiple shifting contingencies. It is these entanglements that social science integrated in clinical trials should be aptly placed to respond to. Yet in spite of equity in funding, management structures, coordination, and a successful collegiate ethos, collaboration in this case did not result in any extrapolation of social science findings to the main trial result; in spite of the inaccuracies uncovered, the CRF remained the source of “objective” data in the final analysis.

RCTs have been recognized as the appropriate research design for determining drug safety and efficacy even by the most critical social scientists. However, social scientists have been vocal in their objection to the use of the RCT in evaluating non-drug interventions, such as health promotion or complex interventions [27].

Vaginal microbicides contain a large dose of both the social and the biomedical, and it is therefore fitting that they be evaluated using RCTs with integrated social science. Large phase III microbicide trials are really studies of effectiveness rather than efficacy; in other words, they measure the effect of the drug in a real-world setting rather than in the controlled conditions of the laboratory or clinical research suite. In these (quasi) real-world settings, the drug does not get used in a vacuum, where efficacy can be measured based on the assumption of use and exposure. Simple causality cannot be inferred. Instead, the drug is used in a complex social world of inequitable power relations between women and men, sexual practices which may affect drug efficacy (such as vaginal douching and insertion) and social norms of commodity sharing and rationing (potentially disrupting drug allocation and reducing adherence if a woman’s gel supply runs low).

Integrating social science methods is indicative of recognition that the experimental method alone is insufficient to measure cause and effect in the social world. It responds directly to charges that the RCT is reductionist and objectivist; the job now is to advance understanding of how social theory and qualitative data can more fundamentally inform the results of RCTs.

We argue that RCTs with well-funded integrated social science may provide more meaningful results (particularly where the result is flat or “negative”) since social science can illuminate the interplay of continuous, uncontrolled and otherwise unmeasured variables potentially determining biological and behavioural endpoints. The solution to the tensions that remain between social and biomedical researchers is not for social scientists to self-censor, believing that collaboration with epidemiologists signals the end of critical and theoretical work, or to abandon applied collaboration all together. In our experience with MDP, we found colleagues receptive to critique, where this was seen as a means of improving the trial. In short, the key was to *engage*, to put social science to the task of questioning what good evidence is, and how we know it when we see it.

## Competing interests

The authors declare that they have no competing interests.

## Authors’ contributions

CM participated in the design of the study, was responsible for its day-to-day coordination, and drafted the manuscript. RP conceived of the study, participated in its coordination, and provided critical input into the manuscript.
